# Expression and prognostic significance of zinc fingers and homeoboxes family members in renal cell carcinoma

**DOI:** 10.1371/journal.pone.0171036

**Published:** 2017-02-02

**Authors:** Ryuk-Jun Kwon, Yun Hak Kim, Dae Cheon Jeong, Myoung-Eun Han, Ji-Young Kim, Liangwen Liu, Jin-Sup Jung, Sae-Ock Oh

**Affiliations:** 1 Departments of Anatomy, School of Medicine, Pusan National University, Yangsan, Republic of Korea; 2 Gene & Therapy Research Center for Vessel-associated Diseases, Pusan National University, Yangsan, Republic of Korea; 3 Department of Statistics, Korea University, Seongbuk-gu, Seoul, Republic of Korea; 4 Departments of Physiology, School of Medicine, Pusan National University, Yangsan, Republic of Korea; Sapporo Ika Daigaku, JAPAN

## Abstract

Zinc fingers and homeoboxes (ZHX) is a transcription repressor family that contains three members; ZHX1, ZHX2, and ZHX3. Although ZHX family members have been associated with the progression of cancer, their values as prognostic factors in cancer patients have been poorly examined. Renal cell carcinoma (RCC) is a highly heterogeneous, aggressive cancer that responds variably to treatment. Thus, prognostic molecular markers are required to evaluate disease progression and to improve the survival. In clear cell RCC (ccRCC), ZHX1 and ZHX3 expression were found to be down-regulated but ZHX2 was up-regulated, and the expressions of ZHX1 and ZHX3 were significantly associated with pathological stage. Furthermore, Kaplan-Meier and multivariate regression analysis showed that reduction in the mRNA expression of ZHX1 was associated with poorer survival. Taken together, the present study shows loss of ZHX1 is correlated with ccRCC progression and suggests it is an independent prognostic marker in ccRCC.

## Introduction

Renal cell carcinoma(RCC) is the most common type of cancer originating from the renal cortex[1], and is classified according to its pathological characteristics. Renal clear cell carcinoma (ccRCC) is the most common subtype;other subtypes include papillary, chromophobe, collecting duct, and unclassified RCC[2]. The incidence rates of RCCs have been steadily increasing at a rate of 2 to 4% per year over past decades[3]. Karnofksy performance status, low level of hemoglobin, elevated platelet count, and elevated corrected calcium are known as risk factors in RCC, and poor risk patients have a 2-year overall survival of only 7%[4]. Furthermore, patients often show poor or partial response to traditional chemotherapy and radiation therapy[5], and thus, despite treatment advances made, RCC remains a highly aggressive and often fatal disease. To improve the poor survival of ccRCC, new therapeutic or prognostic markers need to be developed.

Several molecular prognostic markers have been proposed for RCC[6, 7]. High levels of Ki67 and HIF1A expression in ccRCC were reported to be correlated with poor survival[8, 9], and IMP3-positive ccRCC was found to have a higher risk of metastasis than IMP-negative ccRCC[10]. In addition, the upregulation of FASN, which is involved in fatty acid synthesis, was also reported to be correlated with poor prognosis[11]. These reports demonstrate the identification of prognostic markers associated with the pathogenesis of RCC is required for the evaluation of tumor progression and the personalization of treatment.

The zinc fingers and homeoboxes (ZHX) family contains known nuclear transcription repressors, and is composed of three members, that is,ZHX1, ZHX2, and ZHX3[12]. It has been suggested ZHX family members act as tumor suppressors, although the functions of ZHX3 are still undefined in cancer. ZHX1 overexpression inhibited the proliferation of hepatocellular carcinoma (HCC) cells and its downregulation increased the proliferation of gastric cancer cells [13, 14]. Similarly, ectopic ZHX2 expression reduced HCC cell and tumor xenograft growth in mice[15]. The above-mentioned observations suggest loss of ZHX family members is associated with cancer progression and might be of clinical importance. However, it has been poorly characterized whether the expression of ZHX family members is correlated with stage or prognosis.

In the present study, we compared the expressions levels of ZHX family members in non-tumor tissues and ccRCC tissues, and examined the influence of ZHX family members on stage and overall survival. In addition, we investigated correlation between their expressions and clinical characteristics, and performed univariate and multivariate analysis of prognostic factors for overall survival.

## Materials and methods

### Patients and samples

The Cancer Genome Atlas (TCGA) database stores datasets containing RNA-seq expression and clinical information on various cancers, including ccRCC. To easily analyze the big data, the cBioPortal online platform (http://www.cbioportal.org/) provides small size version of cancer datasets released from TCGA database. In the present study, a TCGA dataset (RNA-seq illumine HighSeq) of 538 ccRCC patients in the cBioPortal platform was initially downloaded (Table 1).

**Table 1:** Patient characteristics of CGA cohort.

Patients characteristics			Total (%)
Histological Diagnosis (n = 521)	Kidney clear cell renal carcinoma		521 (100.0)
Overall survival months (mean±SD, n = 521)			44.36±32.32
Age (mean±SD, n = 521)			60.56±12.09
Sex (n = 521)	Male		334 (64.1)
	Female		187 (35.9)
Ethnicity (n = 521)	Asian		8 (1.5)
	Black or American		54 (10.4)
	White		452 (86.8)
	Unknown		7 (1.3)
T stage (n = 521)	T1 –T2		335 (64.3)
	T3 –T4		186 (35.7)
N stage (n = 521)	N0		232 (44.5)
	N1		14 (2.7)
	Unknown		275 (52.8)
M stage (n = 521)	M0		413 (79.3)
	M1		77 (14.8)
	Unknown		31 (6)
AJCC stage (n = 521)	I		260 (50.0)
	II		57 (10.9)
	III		122 (23.4)
	IV		82 (15.7)
Risk factors	Hemoglobin level (n = 521)	Low *	259 (49.7)
		Normal	180 (34.5)
		Elevated	5 (1.0)
		NA	77 (14.8)
	Platelet count (n = 521)	Low	46 (8.8)
		Normal	353 (67.8)
		Elevated *	36 (6.9)
		NA	86 (16.5)
	Serum calcium level (n = 521)	Low	201 (38.6)
		Normal	147 (28.2)
		Elevated *	10 (1.9)
		NA	163 (31.3)

* High risk factor of ccRCC

The reason why we selected the data is that the number of ccRCC patients in TCGA database is more than in other databases.14 of the 538 ccRCC patients were excluded because clinical information and RNA expression data were not available. Tissues with genetic mutation in ZHX family members were excluded (2 tissues for ZHX1, 3 tissues for ZHX2, 6 tissues for ZHX3). To compare the expressions of ZHX family members in normal tissues and ccRCC tissues, we used GDAC platform(http://gdac.broadinstitute.org/). Data of normal tissues with genetic mutation in ZHX family members were removed from the analysis (1 tissue for ZHX1, 3 tissues for ZHX3).

The Cancer Genome Atlas (TCGA) database contains RNA-seq expression and clinical information of patients with ccRCC, but the size of data is big. To easily analyze the big data, cBioPortal online platform provides small size version of cancer datasets released from TCGA database. So, we downloaded the dataset of 538 ccRCC patients released from TCGA in the cBioPortal platform. The reason why we selected the data is that the number of ccRCC patients in TCGA database is more than in other databases. To analyze more data, we obtained the second cohort of patients with ccRCC from NCBI Gene Expression Omnibus database (GSE3538). Patients’ characteristics were previously described[16].

### Statistical analysis

To compare differences between the expression of ZHX family members in ccRCC and non-tumor tissues, we initially applied the Shapiro-Wilk normality test. If data were normally distributed, the student’s t-test was used, and if not the Mann-Whitney U test (Wilcoxon’s signed rank test)was utilized to determine the significances of intergroup differences. P values of <0.05 were deemed statistically significant. To examine relations between the expressions of ZHX family members and clinical stage, we used the t-test or the Mann-Whitney U test after normality testing. Overall survival was determined from date of diagnosis to death or last follow-up (censored data). To plot Kaplan-Meier curves, patients with ccRCC were divided into low (<median) and high (≥median) expression groups based on the median expression levels of ZHX1-3. The Log-rank test was used to calculate p values. To analyze correlation between the expression of ZHX family members and clinical characteristics, Fisher’s exact test was performed. Multivariate Cox proportional hazards regression analysis was performed to identify significant factors affecting overall survival.

## Results

### Expressions of ZHX family members in ccRCC

**Fig 1:**
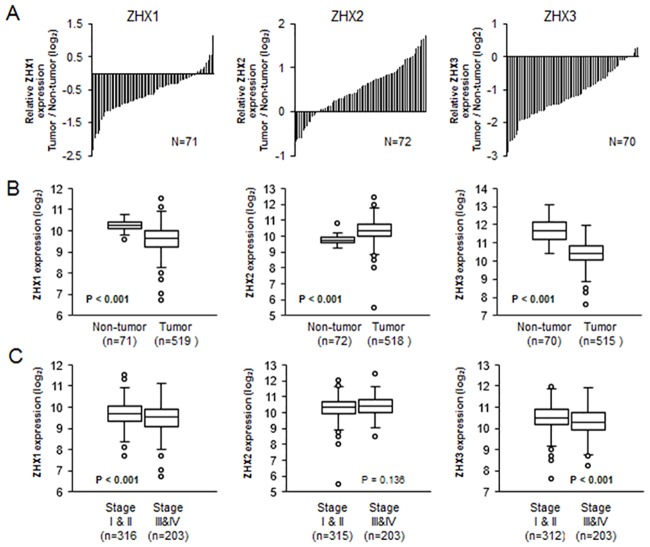
Expressions of ZHX family members and their relationship with clinical stage in ccRCC. (A) Relative expression levels of ZHX family members in ccRCC compared with those in normal tissues, are presented. Expression values of the three ZHX family members (ZHX1-3) in 70 to 72 tumor (ccRCC) tissues were divided by those of paired non-tumor tissues from the same TCGA cohort. (B) The expression levels of ZHX family members in non-tumor and tumor tissues (both paired and not-paired) are presented as a Box-whisker plot. Significances were calculated using the t-test or the Mann-Whitney U test (ZHX1: p < 0.001, ZHX2: p < 0.001, ZHX3: p < 0.001). (C) ZHX1 (stage I&II: n = 316, stage III&IV: n = 203), ZHX2 (stage I&II: n = 315, stage III&IV: n = 203), and ZHX3 (stage I&II: n = 312, stage III&IV: n = 203) expression levels according to ccRCC stage (stages I-II and III-IV) are represented as a Box-whisker plot. Significances of differences were determined using the t-test or the Mann-Whitney U test (ZHX1: p <0.001, ZHX2: p = 0.136, ZHX3: p <0.001).

First, we analyzed RNA-seq data obtained from paired non-tumor and tumor tissues of same patients with ccRCC. To evaluate relative ZHX expression, expression values of ZHX family members in tumor tissues were individually divided by those of paired non-tumor tissues. The expressions of ZHX1 and ZHX3 in most ccRCC tissues were lower than in paired non-tumor tissues, whereas ZHX2 expression was higher in most ccRCC tissues (Fig 1A).

Because not-paired tumor tissues were much more than paired tissues, we compared expressions of ZHX family members using both paired and not-paired tissues. The expressions of ZHX1 (p<0.001) and ZHX3(p<0.001) were significantly lower but that of ZHX2 (p<0.001) was significantly higher in ccRCC tissues than in normal tissues (Fig 1B). Those results indicate that low ZHX1 and ZHX3 expressions and high ZHX2 expression might be associated with the development of ccRCC.

### Association between the expressions of ZHX family members and clinical characteristics

Staging of ccRCC is related to cancer cell proliferation, metastasis, and invasiveness[17]. Thus, to investigate whether ZHX family members are related with ccRCC progression, we evaluated relationships between the expression levels of ZHX family members and clinical stage. Patients were divided into two groups, that is, early stage (I-II) and advanced stage (III-IV). The expression levels of ZHX1 (p<0.001) and ZHX3 (p<0.001) were significantly lower in advanced stage than in early stage (Fig 1C). However, the expression level of ZHX2 (p = 0.136) was higher in advanced stage than in early stage although it was not significant (Fig 1C). Those results indicate that low ZHX1 and ZHX3 expression are associated with the progression of ccRCC.

Next, we examined correlation between the ZHX family members’ expression and other clinicopathological characteristics of patients with ccRCC. The expression levels of ZHX family members were associated with gender of patients (ZHX1, p = 0.013; ZHX2, p = 0.022; ZHX3, p = 0.028; Table 2, S1 and S2 Tables). Moreover, the expression levels of ZHX1 (p = 0.005) and ZHX3 (p<0.001) were associated with T stage and the ZHX1 expression level (p = 0.045) was associated with M stage. However, the expression levels of ZHX family members were not correlated with known risk factors. (Table 2, S1 and S2 Tables).

**Table 2:** Correlation between ZHX1 expression and clinical characteristics in ccRCC.

	ZHX1 expression			
Characteristic	Total N	Low	High	P-value
Age (years)				P = 0.667
< 60	519	123	118	
>60		136	142	
Gender				**P = 0.013**^†^
Male	519	180	153	
Female		79	107	
T stages				**P = 0.005**^†^
T1 –T2	519	151	183	
T3 –T4		108	77	
M stages				**P = 0.045**^†^
M0	488	190	222	
M1		45	31	
N stages				P = 0.598
N0	247	104	128	
N1		8	7	
AJCC stages				**P = 0.002**^†^
Stage I—II	519	140	176	
Stage III—IV		119	84	
Hemoglobin level				P = 0.442
Low *	443	134	124	
Normal, Elevated		89	96	
Platelet count				P = 0.862
Low, Normal	434	199	199	
Elevated *		19	17	
Serum calcium				P = 1.000
Low, Normal	357	176	171	
Elevated *		5	5	

* High risk factor of ccRCC,

^†^ p<0.05

To investigate possible targets associated with ZHX1 and ZHX3 in ccRCC, we analyzed mRNA co-occurrences in cBioPortal platform. The ZHX1 and ZHX3 expressions were significantly and inversely correlated with expressions of VEGFB or USF2 and NMI or ARPC5, respectively (S1 Fig).

### Association between the expression levels of ZHX family members and overall survival

**Fig 2:**
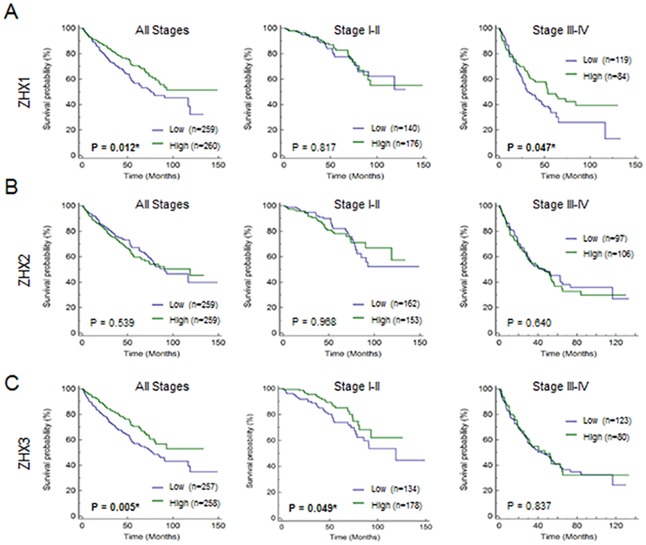
Association between the expression levels of ZHX family members and overall survival. Low expressions of ZHX 1 and 3 were associated with poorer overall survival. Overall survivals for ccRCC patients with low and high expression levels of ZHX family members were represented using Kaplan-Meier curves. Significances of differences were determined using the Log rank test. Left, middle or right three panels present Kaplan-Meier curves for all stages, early stage (I-II) or advanced stage (III-IV) patient group respectively. (A) ZHX1—All stages (low: n = 259, high: n = 260): p = 0.012*, stage I-II (low: n = 140, high: n = 176): p = 0.817, stage III-IV (low: n = 119, high: n = 84): p = 0.047* (B) ZHX2 –All stages (low: n = 259, high: n = 259): p = 0.539, stage I-II (low: n = 162, high: n = 153): p = 0.968, stage III-IV (low: n = 97, high: n = 106): p = 0.640 (C) ZHX3 –All stages (low: n = 257, high: n = 258): p = 0.005*, stage I-II (low: n = 134, high: n = 178): p = 0.049*, stage III-IV (low: n = 123, high: n = 80): 0.837. * p<0.05.

To determine whether the expression levels of ZHX family members in cancer cells affect prognosis of ccRCC patients, Kaplan-Meier curves were plotted for two groups (low and high expression of ZHX family members) against overall survival. Patients with low ZHX1 (p = 0.012) or ZHX3 (p = 0.005) expression were found to show significantly poorer overall survival (Fig 2). However, ZHX2 expression level did not affect overall survival (p = 0.539, Fig 2). To examine whether clinical stage can affect the prognostic significance of ZHX family members, we performed Kaplan-Meier plotting after dividing patients into early and advanced stages. Notably, the association of ZHX1 or ZHX3 with overall survival was significant in advanced (p = 0.047) or early stage respectively (p = 0.005, Fig 2). Next, we examined disease specific survival related to the mortality rate of patients with ccRCC. Kaplan-Meier curves were presented for two groups (low and high expression of ZHX family members) against disease specific survival. Disease specific survival were shorter for patients with low ZHX1 (p = 0.051) or ZHX3 (p = 0.033) expression than those with high ZHX1 or ZHX3 expression (S2 Fig).

To further support our result, we analyzed the second cohort of patients with ccRCC. The data set was obtained from NCBI Gene Expression Omnibus database (GSE3538). Patients’ characteristics were previously described [16]. Kaplan-Meier curves were presented for two groups (low and high expression of ZHX 1–3) against overall survival. Patients with low ZHX2 and ZHX3 expression showed poorer overall survival (S3 Fig, p = 0.0073 for ZHX2, p = 0.0372 for ZHX3). Patients with low ZHX1 expression also showed poorer overall survival although it was not significant (S3 Fig, p = 0.061 for ZHX1).

Next, we examined how expressions of ZHX family members affect mean survival. The mean survival of ccRCC patients were significantly different depending on the expression levels of ZHX1 and ZHX3 (S3 Table). The mean survival in the ZHX1-high group was 99.69 months but that in the ZHX1-low group 78.91 months. The mean survival in the ZHX3-high group was 92.76 months but that in the ZHX3-low group 83.73 months. The median survival in patients with stage III and IV of ccRCC was also significantly different depending on the expression levels of ZHX1 (S4 Table). Those results indicate that loss of ZHX1 and ZHX3 expression in ccRCC could be independent markers of survival.

To further evaluate the prognostic importance of ZHX family expression, we performed univariate analysis and multivariate Cox proportional hazard regression analysis. Age (p<0.001), ZHX1 (p = 0.013), ZHX3 (p = 0.004), and AJCC stage (p<0.001) in univariate analysis were found to be significant indicators of overall survival (Table 3).

**Table 3:** Univariate and multivariate analysis of prognostic factors in patients with ccRCC for overall survival.

	Univariate Analysis p-value	Multivariate analysis (Total N = 510)			
		p-value	Hazard Ratio	CI (Lower 95%)	CI (Upper 95%)
**Age** <60 (vs ≥60)	**P < 0.001**	**0.010**	1.540	1.111	2.136
**Gender** Male (vs female)	P = 0.599	0.718	1.062	0.766	1.472
**ZHX1** Low (vs High)	**P = 0.013**	**0.043**	0.726	0.526	0.990
**ZHX2** Low (vs High)	P = 0.489	0.589	1.088	0.802	1.475
**ZHX3** Low (vs High)	**P = 0.004**	0.235	0.823	0.596	1.135
**Stage** I+II (vs III+IV)	**P < 0.001**	**<0.001**	3.232	2.331	4.482

In multivariate analysis, age (p = 0.010), ZHX1 (p = 0.043), and AJCC stage (p<0.001) were significantly associated with overall survival (Table 3). Hazard ratios of ZHX1 was 0.726, indicating ZHX1 have potential use as independent prognostic markers in ccRCC. In addition, we also compared the prognostic significance of ZHX family members with those of other known prognostic markers (FASN, HIF1A, IMP3 and MKI67) using multivariate regression analysis. The analysis showed that ZHX1 (p = 0.035) and ZHX3 (p = 0.023) were independent prognostic markers for ccRCC together with FASN (S5 Table).

**Fig 3:**
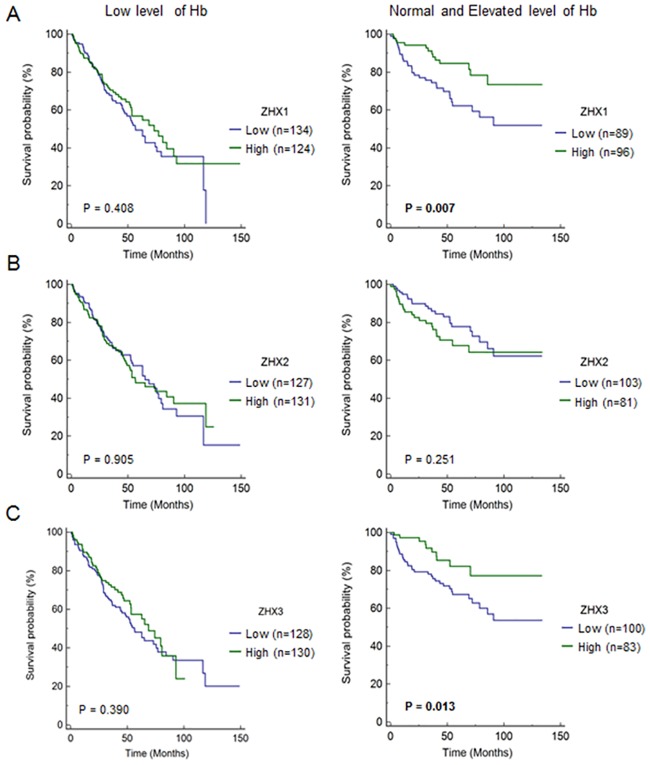
Kaplan-Meier plots of expressions of ZHX family members in subgroup of Hb level. (A-C) Overall survivals for ccRCC patients in low level and normal and elevated level of Hb based on ZHX1-3 expression (ZHX1—low level of Hb (low: n = 134, high: n = 124): p = 0.408, normal and elevated level of Hb (low: n = 89, high: n = 96): p = 0.007; ZHX2—low level of Hb (low: n = 127, high: n = 131): p = 0.905, normal and elevated level of Hb (low: n = 103, high: n = 81): p = 0.251; ZHX3—low level of Hb (low: n = 128, high: n = 130): p = 0.390, normal and elevated level of Hb (low: n = 100, high: n = 83): p = 0.013). Hb: hemoglobin.

There are several representative risk factors in ccRCC which are Karnofksy performance status, low level of hemoglobin, elevated platelet count, and elevated corrected calcium. We examined whether these risk factors can affect the prognostic significance of ZHX family members. The prognostic value of ZHX1 expression was significant in normal/elevated hemoglobin group (p = 0.007), in low/normal platelet group (p = 0.009) and in low/normal serum calcium group (p = 0.037) (Fig 3 and S4 and S5 Figs). The prognostic value of ZHX3 expression was significant in normal/elevated hemoglobin group (p = 0.013) and in low/normal serum calcium group (p = 0.025). However, ZHX2 expression did not affect overall survival irrespective of risk factors.

## Discussion

ZHX family members has been described as tumor suppressor genes in several studies, but their prognostic relevance has been poorly characterized. The present study shows for the first time that ZHX1 and ZHX3 are upregulated and ZHX2 is downregulated and suggests that ZHX1 and ZHX3 be considered independent prognostic markers in ccRCC.

The expressional down-regulations of ZHX family members have been reported in several cancers. In hepatocellular carcinoma and gastric cancer, the expression of ZHX1 was lower than in normal tissues[13, 14], and the nuclear expression of ZHX2 was diminished in HCC[15]. In the present study, the expressions of ZHX1 and ZHX3 were reduced in ccRCC, but the expression of ZHX2 was increased in ccRCC compared to the normal tissues (Fig 1), which suggests the expressions of ZHX family members are cancer type-specific.

In the present study, results of the second cohort (S3 Fig) do not reflect all of results of the first cohort (Fig 2). The results of overall survival of ZHX2 in the first and the second cohorts did not show the consistent trend, which suggests that ZHX2 might not be related with the overall survival of patients with ccRCC. However, the results of overall survival of ZHX1 and ZHX3 in the first and the second cohorts showed the consistent trend although p-value of ZHX1 in the second cohort is not significant. The first cohort, TCGA data (n = 521) includes more numbers of patients than the second cohort (n = 177) when those data were initially obtained. Owing to the difference of numbers of patients between two cohorts, we might not observe significance of ZHX1 in the second cohort.

The expressions of ZHX family members have also been associated with cancer progression and survival time of patients. For example, the reduced ZHX1 expression was associated with TNM stage progression in gastric cancer [14], and a reduction in nuclear ZHX2 expression was associated with short survival in HCC [15]. In the present study, reduction of ZHX1 and ZHX3 expression was found to be associated advanced pathological stage and poor overall survival in ccRCC (Figs 1C and 2). However, ZHX2 expression was not associated with stage and overall survival. The prognostic significance of ZHX1 in ccRCC was further confirmed by multivariate regression analysis (Table 3). Interestingly, the prognostic value of ZHX1 is significant in advanced stage group, normal/elevated hemoglobin group, low/normal platelet group and low/normal serum calcium group. However, the number of patients in elevated platelet group and elevated serum calcium group was relatively small. Therefore, larger scale study needs to be carried out in the future to confirm the result.

The present study showed that ZHX1 expression is correlated with T stages and M stages, and ZHX3 expression is correlated with T stages in ccRCC. However, the underlying mechanism how ZHX1 is correlated with the progression of ccRCC is poorly characterized. ZHX1 has been reported to induce apoptosis and cell cycle arrest (G1/S) by regulating cyclin D1, cyclin E, Bcl2, Bax, and cleaved caspase-3 in gastric cancer[14], and ZHX2 overexpression was correlated with low expressions of cyclin A and cyclin E in HCC[15]. To understand the underlying mechanisms of ZHX1 and ZHX3 during ccRCC progression, we analyzed their mRNA co-occurrence using cBioPortal platform data (S1 Fig). The analysis revealed inverse relationships between the expressions of ZHX1 and ZHX3 and some well-established oncogenes. For example, ZHX1 expression was inversely related with those of USF-2 and VEGF-B, which have been associated with the proliferation of lung cancer cells and endothelial cell migration, respectively[18, 19]. In addition, the expression of ZHX3 was found to be inversely related with the expressions of NMI and ARPC5, which have been associated with proliferation and motility of cancer cells, respectively[20, 21]. More studies need to be carried out to reveal the underlying mechanisms of ZHX1 in ccRCC cells.

## Conclusions

The present study shows expression of ZHX family members is correlated with ccRCC progression and prognosis. The study suggests that ZHX1 is a new independent prognostic marker in ccRCC.

## Supporting information

S1 Fig ZHX1/3 and other targets (VEGFB, USF2, ARPC5, and NMI) were found to be inversely associated with ccRCC progression. Correlation coefficients were calculated by Pearson’s correlation analysis. The expressions of ZHX1 and VEGFB or USF2 were inversely related with correlation coefficients of -0.52 and -0.62 for VEGF-B and USF2 (p <0.001 for both). In addition, the expressions of ZHX3 and NMI or ARPC5 were also inversely related with correlation coefficients of -0.40 for NMI and -0.47 for ARPC5 (p <0.001 for both).
(TIF)

S2 Fig Kaplan-Meier plots for disease specific survival in patients with ccRCC. (A-C) Disease specific survivals for ccRCC patients were presented based on low and high expression of ZHX family members. (ZHX1 (low: n = 203, high: n = 219): p = 0.009; ZHX2 (low: n = 210, high: n = 211): p = 0.820; ZHX3 (low: n = 255, high: n = 162): p = 0.124). (D) The mean and median disease-free survival of patients with ccRCC according to expressions of ZHX family members.
(TIF)

S3 Fig Kaplan-Meier plots for overall survival in the second cohort patients with ccRCC. (A-C)Overall survivals for ccRCC patients were analyzed using the second cohort and the results were presented based on low and high expression of ZHX family members using maximal AUC value. Several statistical tests to analyze survival curve are Log-rank, Gehan-Breslow-Wilcoxon and Fleming-Harrington. Among them, Fleming-Harrington test was used instead of Log-rank test for the ZHX1 analysis of the second cohort. Fleming-Harrington test weights late time, not early time. Because Kaplan-Meier curves for low and high ZHX1 in early time are overlapped, Fleming-Harrington test is more appropriate than Log-rank for the ZHX1 analysis. (ZHX1 (low: n = 40, high: n = 155): p = 0.061; ZHX2 (low: n = 73, high: n = 96): p = 0.0073; ZHX3 (low: n = 28, high: n = 133): p = 0.0372). (D) The mean and median overall survival of patients with ccRCC according to expressions of ZHX family members.
(TIF)

S4 Fig Kaplan-Meier plots of expressions of ZHX family members in subgroup of platelet count. (A-C) Overall survivals for ccRCC patients in low and normal count and elevated count of platelet based on ZHX1-3 expression (ZHX1 –low and normal count of platelet (low: n = 199, high: n = 199): p = 0.009, elevated count of platelet (low: n = 19, high: n = 17): p = 0.914; ZHX2—low and normal count of platelet (low: n = 203, high: n = 194): p = 0.248, elevated count of platelet (low: n = 21, high: n = 15): p = 0.820; ZHX3—low and normal count of platelet (low: n = 201, high: n = 195): p = 0.200, elevated count of platelet (low: n = 24, high: n = 12): p = 0.124).
(TIF)

S5 Fig Kaplan-Meier plots of expressions of ZHX family members in subgroup of serum calcium level. (A-C) Overall survivals for ccRCC patients in low and normal level and elevated level of serum calcium based on ZHX1-3 expression (ZHX1 –low and normal level of serum calcium (low: n = 176, high: n = 171): p = 0.037, elevated level of serum calcium (low: n = 5, high: n = 5): p = 0.778; ZHX2—low and normal level of serum calcium (low: n = 176, high: n = 170): p = 0.296, elevated level of serum calcium (low: n = 3, high: n = 7): p = 0.690; ZHX3—low and normal level of serum calcium (low: n = 171, high: n = 175): p = 0.025, elevated level of serum calcium (low: n = 7, high: n = 3): p = 0.579).
(TIF)

S1 Table Correlation between ZHX2 expression and clinical characteristics in ccRCC. (DOCX)

S2 Table Correlation between ZHX3 expression and clinical characteristics in ccRCC. (DOCX)

S3 Table Mean and median survival of patients with ccRCC according to expressions of ZHX family members. (DOCX)

S4 Table Mean and median survival of patients with ccRCC in early and advanced stages. (DOCX)

S5 Table Univariate and multivariate analysis of prognostic markers in patients with ccRCC for overall survival. (DOCX)
