# Supplementary figures and images for: Expression and prognostic significance of zinc fingers and homeoboxes family members in renal cell carcinoma
Source: PLoS One. 2017 Feb 2;12(2):e0171036. doi: 10.1371/journal.pone.0171036 (PMC5289508; doi:10.1371/journal.pone.0171036)

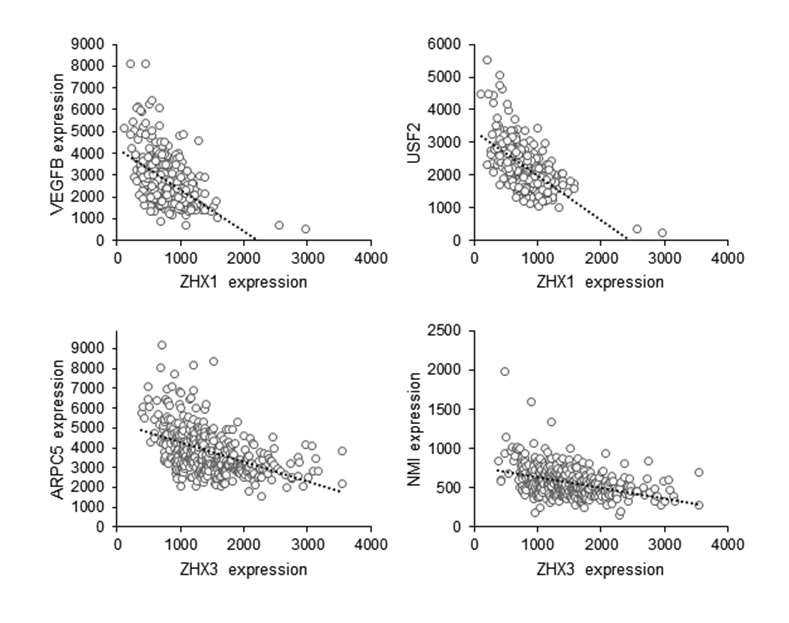

Supplement: S1 Fig — Correlation coefficients were calculated by Pearson’s correlation analysis. The expressions of ZHX1 and VEGFB or USF2 were inversely related with correlation coefficients of -0.52 and -0.62 for VEGF-B and USF2 (p <0.001 for both). In addition, the expressions of ZHX3 and NMI or ARPC5 were also inversely related with correlation coefficients of -0.40 for NMI and -0.47 for ARPC5 (p <0.001 for both). (TIF) [file pone.0171036.s001.tif]

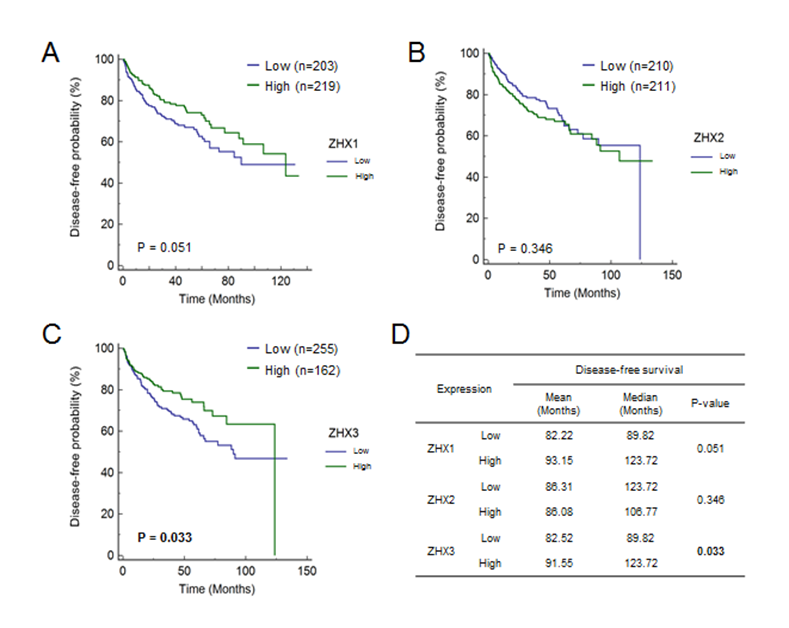

Supplement: S2 Fig — (A-C) Disease specific survivals for ccRCC patients were presented based on low and high expression of ZHX family members. (ZHX1 (low: n = 203, high: n = 219): p = 0.009; ZHX2 (low: n = 210, high: n = 211): p = 0.820; ZHX3 (low: n = 255, high: n = 162): p = 0.124). (D) The mean and median disease-free survival of patients with ccRCC according to expressions of ZHX family members. (TIF) [file pone.0171036.s002.tif]

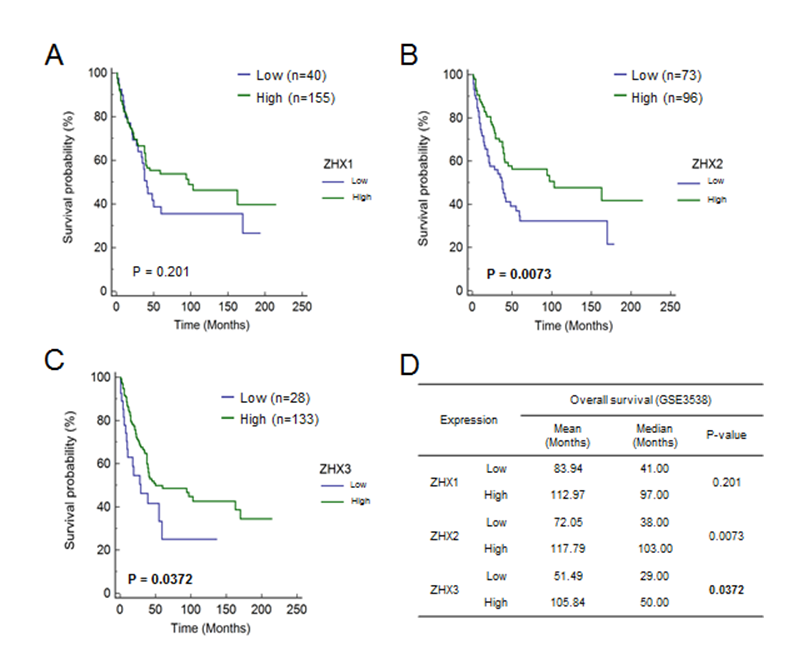

Supplement: S3 Fig — (A-C)Overall survivals for ccRCC patients were analyzed using the second cohort and the results were presented based on low and high expression of ZHX family members using maximal AUC value. Several statistical tests to analyze survival curve are Log-rank, Gehan-Breslow-Wilcoxon and Fleming-Harrington. Among them, Fleming-Harrington test was used instead of Log-rank test for the ZHX1 analysis of the second cohort. Fleming-Harrington test weights late time, not early time. Because Kaplan-Meier curves for low and high ZHX1 in early time are overlapped, Fleming-Harrington test is more appropriate than Log-rank for the ZHX1 analysis. (ZHX1 (low: n = 40, high: n = 155): p = 0.061; ZHX2 (low: n = 73, high: n = 96): p = 0.0073; ZHX3 (low: n = 28, high: n = 133): p = 0.0372). (D) The mean and median overall survival of patients with ccRCC according to expressions of ZHX family members. (TIF) [file pone.0171036.s003.tif]

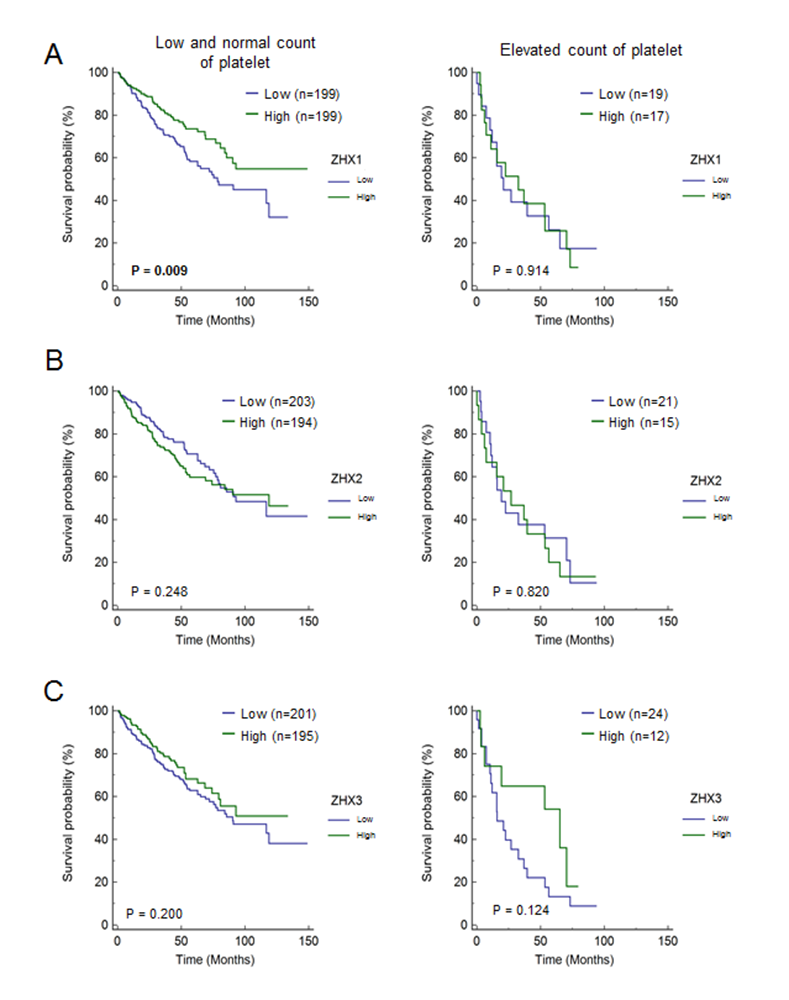

Supplement: S4 Fig — (A-C) Overall survivals for ccRCC patients in low and normal count and elevated count of platelet based on ZHX1-3 expression (ZHX1 –low and normal count of platelet (low: n = 199, high: n = 199): p = 0.009, elevated count of platelet (low: n = 19, high: n = 17): p = 0.914; ZHX2—low and normal count of platelet (low: n = 203, high: n = 194): p = 0.248, elevated count of platelet (low: n = 21, high: n = 15): p = 0.820; ZHX3—low and normal count of platelet (low: n = 201, high: n = 195): p = 0.200, elevated count of platelet (low: n = 24, high: n = 12): p = 0.124). (TIF) [file pone.0171036.s004.tif]

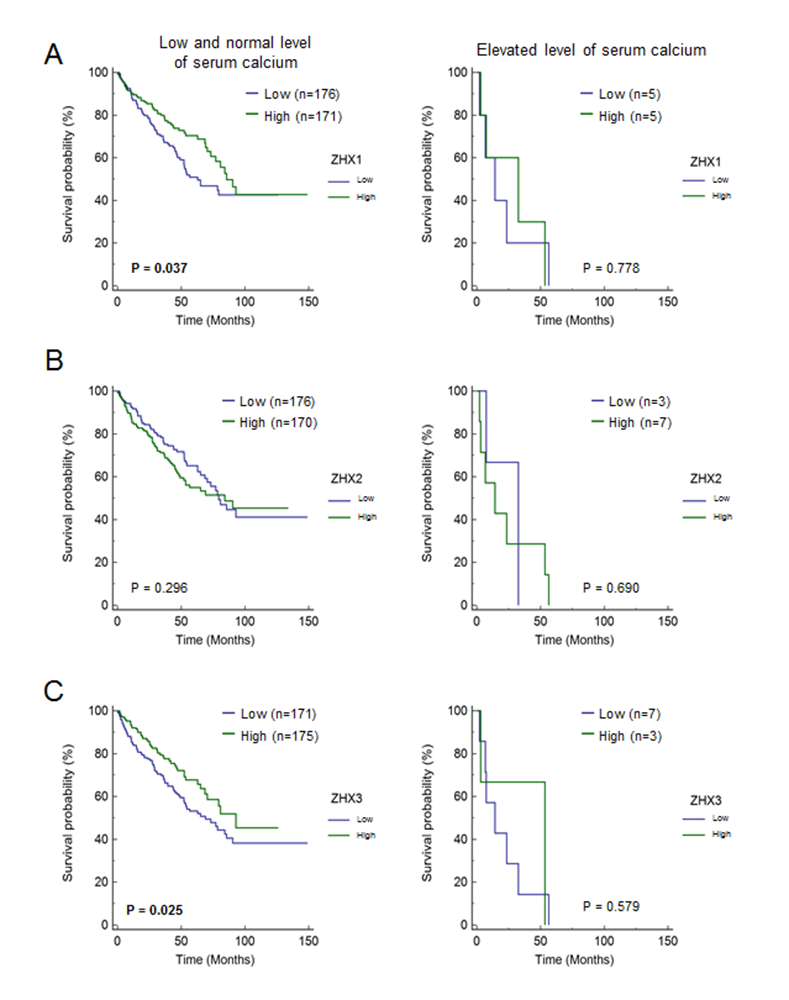

Supplement: S5 Fig — (A-C) Overall survivals for ccRCC patients in low and normal level and elevated level of serum calcium based on ZHX1-3 expression (ZHX1 –low and normal level of serum calcium (low: n = 176, high: n = 171): p = 0.037, elevated level of serum calcium (low: n = 5, high: n = 5): p = 0.778; ZHX2—low and normal level of serum calcium (low: n = 176, high: n = 170): p = 0.296, elevated level of serum calcium (low: n = 3, high: n = 7): p = 0.690; ZHX3—low and normal level of serum calcium (low: n = 171, high: n = 175): p = 0.025, elevated level of serum calcium (low: n = 7, high: n = 3): p = 0.579). (TIF) [file pone.0171036.s005.tif]
