# Supplementary material for: Expression and prognostic significance of zinc fingers and homeoboxes family members in renal cell carcinoma
Source: PLoS One. 2017 Feb 2;12(2):e0171036. doi: 10.1371/journal.pone.0171036 (PMC5289508; doi:10.1371/journal.pone.0171036)
Supplement: S1 Table — (DOCX) [file pone.0171036.s006.docx]

| * High risk factor of ccRCC | ZHX2 expression | | |  |
| --- | --- | --- | --- | --- |
| Characteristic | Total N | Low | High | P-value |
| Age (years) |  |  |  | P = 0.290 |
| < 60 | 518 | 126 | 113 |  |
| >60 |  | 133 | 146 |  |
| Gender |  |  |  | **P = 0.022** |
| Male | 518 | 154 | 180 |  |
| Female |  | 105 | 79 |  |
| T stages |  |  |  | P = 0.199 |
| T1 – T2 | 518 | 174 | 159 |  |
| T3 – T4 |  | 85 | 100 |  |
| M stages |  |  |  | P = 0.620 |
| M0 | 487 | 206 | 204 |  |
| M1 |  | 36 | 41 |  |
| N stages |  |  |  | P = 0.785 |
| N0 | 245 | 116 | 115 |  |
| N1 |  | 8 | 6 |  |
| AJCC stages |  |  |  | P=0.472 |
| Stage I - II | 518 | 162 | 153 |  |
| Stage III - IV |  | 97 | 106 |  |
| Hemoglobin level |  |  |  | P = 0.334 |
| Low * | 442 | 127 | 131 |  |
| Normal, Elevated |  | 103 | 81 |  |
| Platelet count |  |  |  | P = 0.487 |
| Low, Normal | 433 | 203 | 194 |  |
| Elevated * |  | 21 | 15 |  |
| Serum calcium |  |  |  | P = 0.218 |
| Low, Normal | 356 | 176 | 170 |  |
| Elevated * |  | 3 | 7 |  |

* High risk factor of ccRCC
