# Supplementary material for: Expression and prognostic significance of zinc fingers and homeoboxes family members in renal cell carcinoma
Source: PLoS One. 2017 Feb 2;12(2):e0171036. doi: 10.1371/journal.pone.0171036 (PMC5289508; doi:10.1371/journal.pone.0171036)
Supplement: S2 Table — (DOCX) [file pone.0171036.s007.docx]

|  | ZHX3 expression | | |  |
| --- | --- | --- | --- | --- |
| Characteristic | Total N | Low | High | P-value |
| Age (years) |  |  |  | P = 0.427 |
| < 60 | 515 | 114 | 124 |  |
| >60 |  | 143 | 134 |  |
| Gender |  |  |  | **P = 0.028** |
| Male | 515 | 152 | 177 |  |
| Female |  | 105 | 81 |  |
| T stages |  |  |  | **P < 0.001** |
| T1 – T2 | 515 | 146 | 183 |  |
| T3 – T4 |  | 111 | 75 |  |
| M stages |  |  |  | P = 0.080 |
| M0 | 484 | 200 | 208 |  |
| M1 |  | 46 | 30 |  |
| N stages |  |  |  | P = 0.592 |
| N0 | 244 | 129 | 100 |  |
| N1 |  | 10 | 5 |  |
| AJCC stages |  |  |  | **P < 0.001** |
| Stage I - II | 515 | 134 | 178 |  |
| Stage III - IV |  | 123 | 80 |  |
| Hemoglobin level |  |  |  | P = 0.334 |
| Low * | 441 | 128 | 130 |  |
| Normal, Elevated |  | 100 | 83 |  |
| Platelet count |  |  |  | P = 0.081 |
| Low, Normal | 432 | 201 | 195 |  |
| Elevated * |  | 24 | 12 |  |
| Serum calcium |  |  |  | P = 0.337 |
| Low, Normal | 356 | 171 | 175 |  |
| Elevated * |  | 7 | 3 |  |
