# Supplementary material for: Expression and prognostic significance of zinc fingers and homeoboxes family members in renal cell carcinoma
Source: PLoS One. 2017 Feb 2;12(2):e0171036. doi: 10.1371/journal.pone.0171036 (PMC5289508; doi:10.1371/journal.pone.0171036)
Supplement: S3 Table — (DOCX) [file pone.0171036.s008.docx]

| Expression | | Overall survival | | |
| --- | --- | --- | --- | --- |
|  |  | Mean (Months) | Median (Months) | P-value |
| ZHX1 | Low | 78.91 | 75.53 | **0.012** |
|  | High | 99.69 | N.A.* |  |
| ZHX2 | Low | 91.92 | 90.80 | 0.539 |
|  | High | 85.07 | 118.76 |  |
| ZHX3 | Low | 83.73 | 76.97 | **0.005** |
|  | High | 92.76 | N.A.* |  |

* N.A.: not available (no estimated median)
