# Supplementary material for: Expression and prognostic significance of zinc fingers and homeoboxes family members in renal cell carcinoma
Source: PLoS One. 2017 Feb 2;12(2):e0171036. doi: 10.1371/journal.pone.0171036 (PMC5289508; doi:10.1371/journal.pone.0171036)
Supplement: S4 Table — (DOCX) [file pone.0171036.s009.docx]

| Expression | | Stage I and II | | | Stage III and IV | | |
| --- | --- | --- | --- | --- | --- | --- | --- |
|  |  | Mean (Months) | Median (Months) | P-value | Mean (Months) | Median (Months) | P-value |
| ZHX1 | Low | 100.56 | N.A* | 0.817 | 53.03 | 30.62 | 0.047 |
|  | High | 110.46 | N.A* |  | 69.70 | 53.38 |  |
| ZHX2 | Low | 108.69 | N.A* | 0.968 | 64.12 | 43.92 | 0.640 |
|  | High | 104.26 | N.A* |  | 59.49 | 47.04 |  |
| ZHX3 | Low | 102.95 | 118.76 | 0.049 | 57.99 | 43.92 | 0.837 |
|  | High | 103.30 | N.A* |  | 61.24 | 47.04 |  |

* N.A.: not available (no estimated median)
