# Supplementary material for: Expression and prognostic significance of zinc fingers and homeoboxes family members in renal cell carcinoma
Source: PLoS One. 2017 Feb 2;12(2):e0171036. doi: 10.1371/journal.pone.0171036 (PMC5289508; doi:10.1371/journal.pone.0171036)
Supplement: S5 Table — (DOCX) [file pone.0171036.s010.docx]

|  | Univariate Analysis p-value | Multivariate analysis (N=494) | | | |
| --- | --- | --- | --- | --- | --- |
|  |  | p-value | Hazard Ratio | CI (Lower 95%) | CI (Upper 95%) |
| ZHX1 Low (vs High) | P=0.011 | 0.035 | 0.705 | 0.510 | 0.976 |
| ZHX2 Low (vs High) | P=0.572 | 0.802 | 0.961 | 0.703 | 1.313 |
| ZHX3 Low (vs High) | P=0.007 | 0.023 | 0.685 | 0.495 | 0.949 |
| FASN Low (vs High) | P<0.001 | <0.001 | 2.396 | 1.717 | 3.345 |
| HIF1A Low (vs High) | P=0.229 | 0.193 | 1.242 | 0.896 | 1.721 |
| IMP3 Low (vs High) | P=0.548 | 0.909 | 0.980 | 0.698 | 1.377 |
| MKI67 Low (vs High) | P=0.005 | 0.051 | 1.390 | 0.998 | 1.936 |
